# The Human Ocular Surface Microbiome and Its Associations with the Tear Proteome in Dry Eye Disease

**DOI:** 10.3390/ijms241814091

**Published:** 2023-09-14

**Authors:** Irina Schlegel, Claire M. F. De Goüyon Matignon de Pontourade, Joel-Benjamin Lincke, Irene Keller, Martin S. Zinkernagel, Denise C. Zysset-Burri

**Affiliations:** 1 Department of Ophthalmology, Inselspital, Bern University Hospital, University of Bern, 3010 Bern, Switzerland; irina_schlegel@hotmail.com (I.S.); claire.degouyon@insel.ch (C.M.F.D.G.M.d.P.); joel-benjamin.lincke@insel.ch (J.-B.L.); martin.zinkernagel@insel.ch (M.S.Z.); 2 Department for BioMedical Research, University of Bern, 3010 Bern, Switzerland; irene.keller@unibe.ch; 3 Interfaculty Bioinformatics Unit and Swiss Institute of Bioinformatics, University of Bern, 3012 Bern, Switzerland

**Keywords:** chromatography–tandem mass spectrometry, dry eye disease, ocular surface microbiome, tear proteome, whole-metagenome shotgun sequencing

## Abstract

Although dry eye disease (DED) is one of the most common ocular surface diseases worldwide, its pathogenesis is incompletely understood, and treatment options are limited. There is growing evidence that complex interactions between the ocular surface microbiome (OSM) and tear fluid constituents, potentially leading to inflammatory processes, are associated with ocular surface diseases such as DED. In this study, we aimed to find unique compositional and functional features of the OSM associated with human and microbial tear proteins in patients with DED. Applying whole-metagenome shotgun sequencing of forty lid and conjunctival swabs, we identified 229 taxa, with Actinobacteria and Proteobacteria being the most abundant phyla and Propionibacterium acnes the dominating species in the cohort. When DED patients were compared to controls, the species Corynebacterium tuberculostearicum was more abundant in conjunctival samples, whereas the family Propionibacteriaceae was more abundant in lid samples. Functional analysis showed that genes of L-lysine biosynthesis, tetrapyrrole biosynthesis, 5-aminoimidazole ribonucleotide biosynthesis, and the super pathway of L-threonine biosynthesis were enriched in conjunctival samples of controls. The relative abundances of Acinetobacter johnsonii correlated with seven human tear proteins, including mucin-16. The three most abundant microbial tear proteins were the chaperone protein DnaK, the arsenical resistance protein ArsH, and helicase. Compositional and functional features of the OSM and the tear proteome are altered in patients with DED. Ultimately, this may help to design novel interventional therapeutics to target DED.

## 1. Introduction

**Figure 1:**
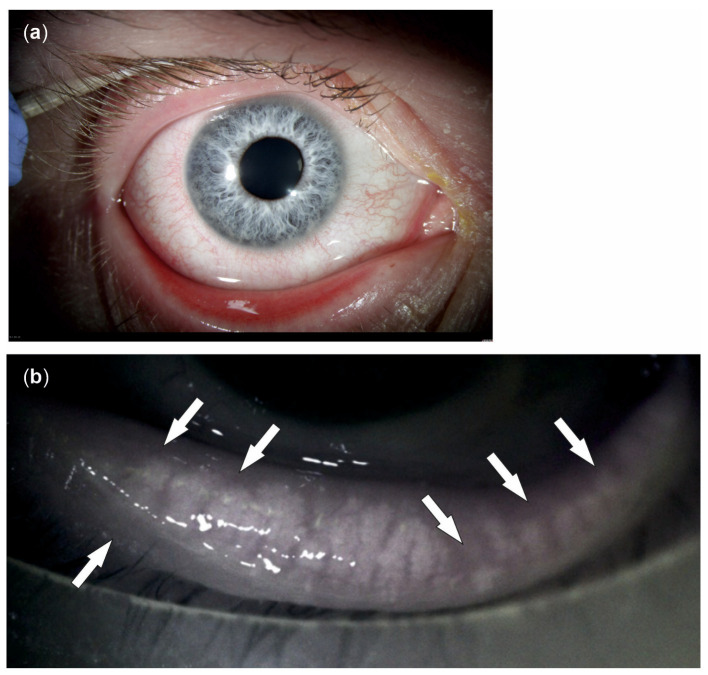
Symptoms and causes of dry eye disease (DED). There is a wide range of symptoms of DED, including irritation, redness, blurry vision, tearing, light sensitivity, and mucus secretion (**a**). Amongst other factors, meibomian gland dysfunction is a leading cause of DED, disrupting the tear film lipid layer by affecting the rate of tear evaporation. A specific meibomian gland feature often observed in patients with DED is shortened glands, i.e., glands that do not extend to their full normal length (arrows in the meibography (**b**)).

Dry eyes and dry eye disease (DED) are considered one of the most common ocular surface diseases worldwide, especially in people of advanced age, who have a prevalence of up to 34% [1,2,3]. DED is characterized by ocular discomforts such as foreign body sensation, burning sensation, itching, or chronic eye pain. It is also associated with reduced and/or fluctuating vision, which may restrict activities of daily living such as driving, reading, or watching television [4]. It is considered a serious public health issue and is associated with a large economic burden [2], both in terms of direct and indirect healthcare costs. Despite this, the current understanding of the pathogenesis of DED is incomplete, and treatment options are limited to moisturizing eye drops, antibiotics, and crude immune modulators with partially severe side effects. In 2017, the TFOS DEWS II Definition and Classification Subcommittee aimed to create an evidence-based definition and classification system for DED. This led to the definition of a multifactorial ocular surface disease, with symptoms originating from tear film instability and hyperosmolarity, ocular surface inflammation and damage, and neurosensory abnormalities (Figure 1) [5].

**Figure 2:**
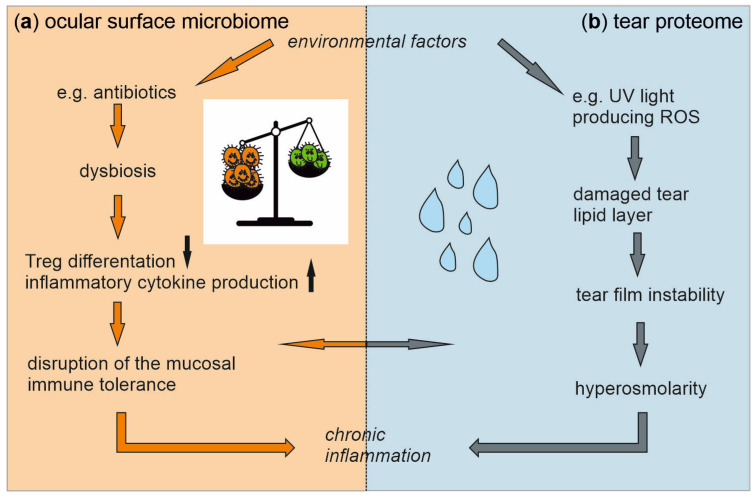
An imbalance in the ocular surface microbiome and the tear proteome, triggered by environmental factors, contributes to dry eye disease (DED) by inducing inflammatory processes. Antibiotics promote an imbalanced state in the ocular surface microbiome. This dysbiosis disrupts mucosal immune tolerance via enhanced inflammatory cytokine production and decreased regulatory T cell (Treg) differentiation, ultimately resulting in inflammation (**a**). Reactive oxygen species (ROS) damage the tear lipid layer, leading to tear film instability and increased osmolarity that induces inflammation (**b**). The induced chronic inflammatory processes are probably a key component in the pathogenesis of DED. Associations between the two systems, i.e., potential interactions between the ocular surface microbiome and the tear proteome in DED, have not been investigated so far and are the subject of this study.

The ocular surface is continuously exposed to irritants, allergens, and pathogens, against which it can mount a prompt immune response resulting in inflammation. Since inflammatory processes result in damaged tissue associated with sight-threatening impairment of the ocular surface, including DED, the mucosal immune system of the ocular surface features a kind of unresponsiveness to potentially harmful antigens called immune tolerance [6]. In the basal state, i.e., in the absence of an infection, dendritic cells with an imprinted tolerogenic profile migrate to the lymph nodes where they present antigens to naïve T cells, thus inducing a regulatory T cell (Treg) phenotype. These Treg cells can circulate to the ocular surface, delivering inhibitory signals, effectively suppressing the local inflammatory responses, and contributing to local homeostasis. However, environmental factors such as antibiotics disturb the homeostatic ecosystem of microbes at the ocular surface, resulting in an unbalanced state called dysbiosis. Many studies show associations between dysbiosis of the intestinal microbiome and multiple eye diseases, including age-related macular degeneration [7,8], retinal artery occlusion [9], and DED [10]. In addition to the influence on the severity of DED [11], intestinal dysbiosis may result in the infiltration of pro-inflammatory factors into the tissues of the ocular surface by mediating autoimmune responses [12,13,14,15,16,17], thus playing an important role in immune homeostasis of the ocular surface [10]. A previous study characterized the ocular surface microbiome taxonomically and functionally [18]. The composition of this microbiome may play an essential role in the induction of inflammatory processes inducing DED via disruption of the ocular immune tolerance (Figure 2a).

Furthermore, environmental factors such as UV light lead to the generation of reactive oxygen species (ROS) that damage the tear lipid layer [19]. By quantifying the human tear proteome, we previously identified tear proteins with an antioxidative effect, such as lactoferrin and S100A proteins [18]. An imbalance between these antioxidative proteins and ROS favors the development of tear film instability [19]. Together, this imbalance may result in tear hyperosmolarity, a hallmark of DED, by inducing pro-inflammatory pathways [20] (Figure 2b).

Since both the microbes at the ocular surface and the tear proteins could be key features in the pathogenesis of DED by inducing inflammatory processes (Figure 2), in this study, we characterized the ocular surface microbiome as well as the tear proteome of patients with DED compared to healthy controls. Moreover, association studies were performed between the ocular surface microbiome and the tear proteome and the metadata used for group assignment to identify the factors leading to the chronic inflammatory processes observed in DED. This approach allows for identifying the factors decisively contributing to DED and associations between DED and the microbiome. Therefore, the results may set a basis for identifying new therapeutic targets for DED and perhaps for associated eye diseases.

## 2. Results

### 2.1. Demographic Data

In total, 20 lid and 20 conjunctival swabs were sequenced from 20 eyes of 10 participants suffering from DED compared to 10 participants with no signs of DED. The two groups were similar in sex and age at the time of collection (Table 1).

### 2.2. Taxonomical and Functional Characterization of the Ocular Surface Microbiome in Dry Eye Disease

**Figure 3:**
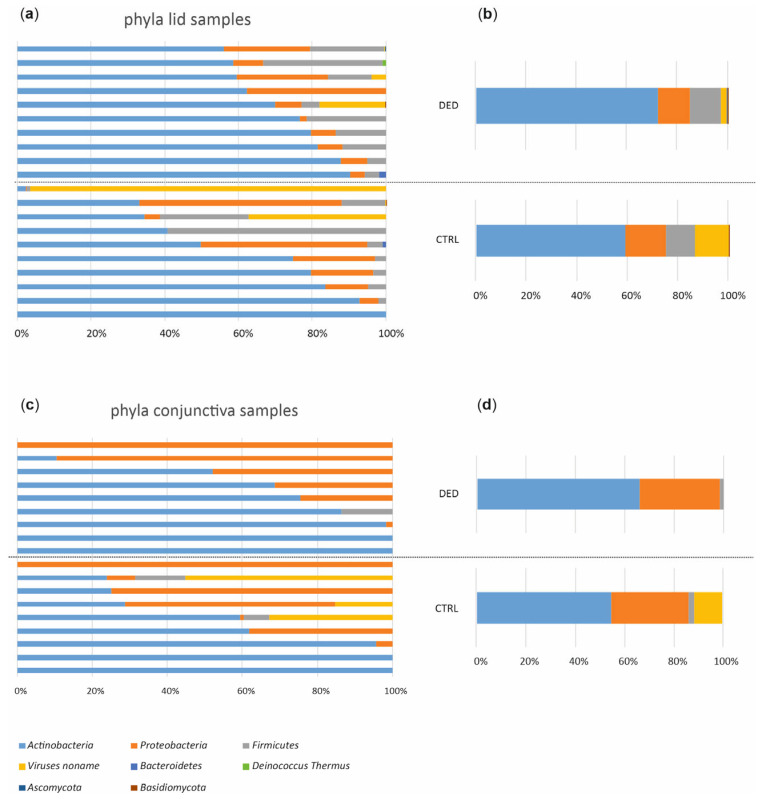
Taxonomic characterization of the ocular surface microbiome. Relative microbiota abundances at the phylum level from lid samples in all study subjects (**a**) and averaged for study groups (**b**). Relative microbiota abundances at the phylum level from conjunctival samples in all study subjects (**c**) and averaged for study groups (**d**). CTRL, control (*n* = 10); DED, dry eye disease (*n* = 10).

Because sequencing libraries from negative controls failed quality and quantity controls for sequencing, it could be assumed that no contaminations were sequenced. In total, 1.5 billion 100 bp paired-end reads with an average insert size of 350 bp were generated, with an average of 36.6 ± 7.8 (s.d.) million reads per sample. Most of these reads were of human origin, as expected and described in previous studies. After trimming and filtering, we kept about 96.4 million non-human, high-quality reads with an average of 2.4 million reads per sample. We identified 229 taxa, most bacteria (93.2%). While the phyla Actinobacteria (64.8%) and Proteobacteria (23.4%) dominated the ocular microbiome composition (Figure 3), Actinobacteria (63.1%) was the most abundant class, and Propionibacterium (31.9%), Agrobacterium (22.4%), and Corynebacterium (21.2%) were the most abundant genera in the cohort. The dominating species, Propionibacterium acnes, was found in 75% of the samples.

**Figure 4:**
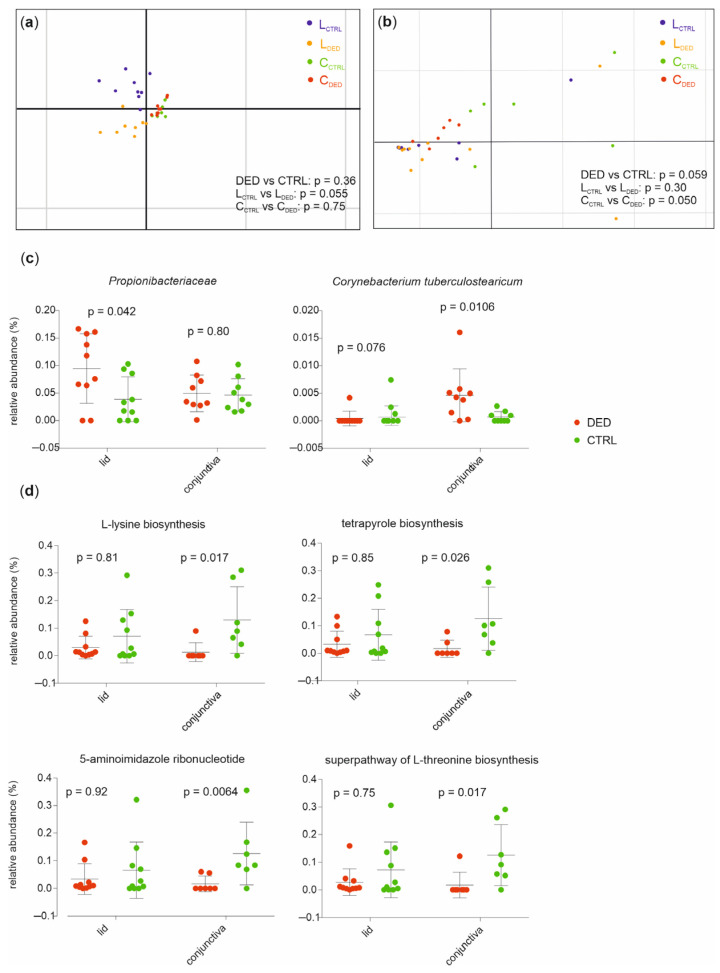
The distinct taxonomical and functional composition of the ocular surface microbiome between DED patients and healthy controls. (**a**) The principal component analysis (PCA) of taxonomical feature abundance did not individually separate DED patients from controls in all samples (*p* = 0.36) or lid samples (*p* = 0.055) and conjunctival samples (*p* = 0.75). (**b**) The PCA of functional feature abundance did not individually separate DED patients from controls in all samples (*p* = 0.059) or lid samples (*p* = 0.30). However, in conjunctival samples, the PCA of functional feature abundance separated patients from controls (*p* = 0.050, PERMANOVA, *n* repeat = 10,000). The relative abundances of taxa (**c**) and pathways (**d**) associated with DED (Mann–Whitney test). Mean values and standard deviations are shown.

When applying a principal component analysis (PCA) with the health status as a grouping variable, the DED group could not be separated from the controls based on differences in microbial abundances (for lid samples *p* = 0.055, conjunctival samples *p* = 0.75; PERMANOVA analysis where the *n* repeat = 10,000, Figure 4a). However, using PCA, the DED group was separated from healthy controls based on differences in relative abundances of functional profiles from lid samples (*p* = 0.050) but not from conjunctival samples (*p* = 0.30; PERMANOVA analysis where the *n* repeat = 10,000, Figure 4b).

To further examine features of the ocular surface microbiome in DED patients, we compared the relative abundances of microbial taxa between patients and controls, demonstrating that in conjunctival samples, the species Corynebacterium tuberculostearicum (*p* = 0.0106) was more abundant in DED patients compared to controls, whereas in lid samples, the family Propionibacteriaceae (*p* = 0.042) was more abundant in patients compared to controls (Welch’s *t*-test, Figure 4c). Furthermore, ocular microbiomes of conjunctival samples from controls were enriched in genes of L-lysine biosynthesis (*p* = 0.017), tetrapyrrole biosynthesis (*p* = 0.026), 5-aminoimidazole ribonucleotide biosynthesis (*p* = 0.0064), and of the super pathway of L-threonine biosynthesis (*p* = 0.017, Mann–Whitney test, Figure 4d).

### 2.3. Associations between the Ocular Surface Microbiome and Clinical Metadata

We used multivariate association by linear models (MaAsLin) to examine whether relative abundances of microbial taxa and pathways were associated with clinical metadata used for group assignment. This approach ensures that only factors associated with the given feature are included in the model, implying that all associations found have been corrected for all other confounding factors. 

Although further investigation is needed, when applying this model to the functional features, it seems that L-arginine biosynthesis in the conjunctiva increases in patients with tear hyperosmolarity (Table 2). For taxonomic features, however, no associations were found with the metadata, neither in the lid nor in conjunctival samples. 

### 2.4. Functional Classification of the Tear Proteome in Dry Eye Disease

**Figure 5:**
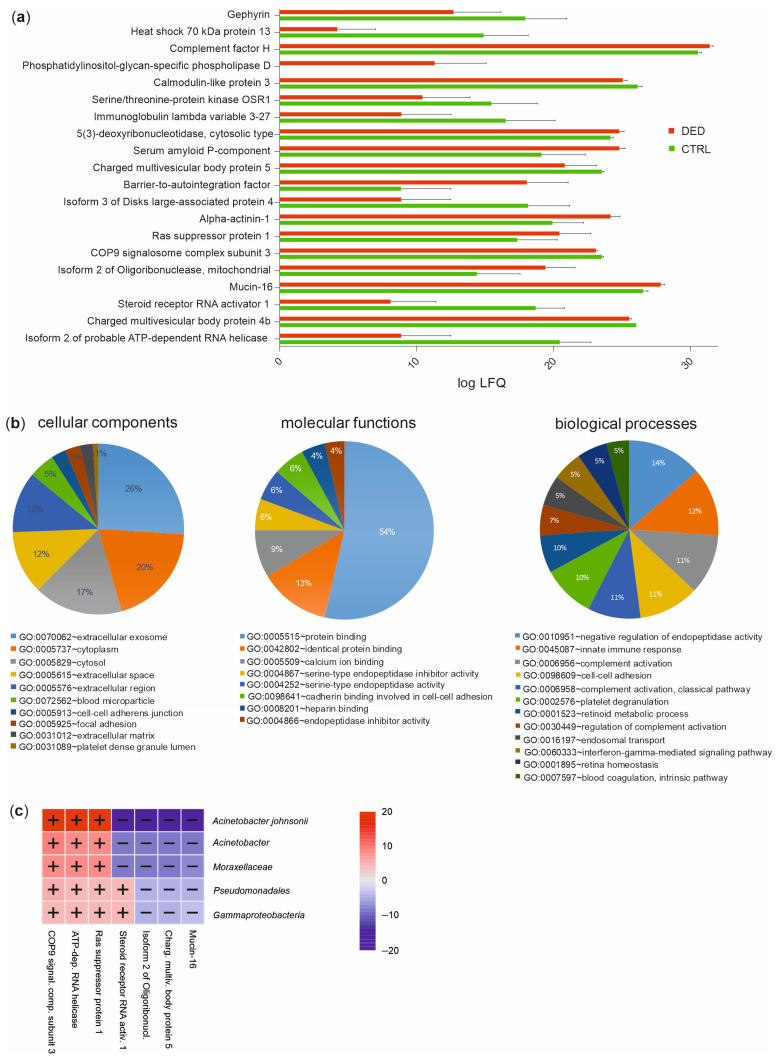
Distinct tear proteome between DED patients and healthy controls. (**a**) Label-free quantification (LFQ) values of the top 20 tear proteins between DED patients and controls. The mean of log10 values and standard deviations are shown. CTRL, control (*n* = 10); DED, dry eye disease (*n* = 10). (**b**) Functional classification of the significantly different tear proteins in DED patients compared to controls based on the Gene Ontology (GO) categories cellular components, molecular functions, and biological processes (DAVID bioinformatics tool). (**c**) Significant associations between taxonomic features of the ocular surface microbiome and the top 20 significantly different tear proteins between DED patients and controls (−log(qval)×ign(coeff), MaAsLin2).

In a previous study, we quantified 2172 human protein groups that were mainly involved in the following biological processes: cell–cell adhesion, proteolysis, oxidation-reduction process, antimicrobial humoral response, and innate immune response according to Gene Ontology (GO) term analysis [18]. Among this tear proteome, 112 proteins were different in quantity between DED patients and controls (Welch’s *t*-test, *p* ≤ 0.05) (according to the mean of the across-samples normalized MaxQuant calculated protein group label-free quantification (LFQ) values). Using the DAVID bioinformatics tool [21,22], functional classification revealed that these proteins are mainly involved in the molecular function of “protein binding” (54%) as well as in biological processes such as “negative regulation of endopeptidase activity” (14%), “innate immune response” (12%), and “complement activation” (11%). These activities mostly take place in the cellular components “extracellular exosome” (26%), “cytoplasm” (20%), and “cytosol” (17%) (Figure 5b). The top 20 proteins between the case and controls (Figure 5a) were used for association studies between the tear proteome and the ocular microbiome. We applied MaAsLin to examine whether relative abundances of the microbiome’s taxonomic and/or functional features were associated with the quantities of the tear proteins. This approach revealed a positive correlation between the steroid receptor RNA activator 1 with the two pathways L-valine biosynthesis and pyrimidine deoxyribonucleotides de novo biosynthesis ll. For taxonomic features, correlations between the class Gammaproteobacteria and lower taxonomic ranks, down to the species Acinetobacter johnsonii, and the following tear proteins were found: COP9 signalosome complex subunit 3, ATP-dependent RNA helicase, Ras suppressor protein, steroid receptor RNA activator, isoform 2 of oligoribonuclease, charged multivesicular body protein, and mucin-16 (Figure 5c).

To further assess the role of the ocular microbiome in host physiology, we identified tear proteins derived from ocular surface microbes. Bacterial tear proteins from Acinetobacter johnsonii, Acinetobacter sp., Agrobacterium radiobacter, Agrobacterium sp., Campylobacter ureolyticus, Corynebacterium accolens, and Cutibacterium acnes were detected. The three most abundant proteins were the chaperone protein DnaK, the arsenical resistance protein ArsH, and helicase (Table 3).

## 3. Discussion

DED is a multifactorial ocular surface disease with symptoms originating from tear film instability and hyperosmolarity, ocular surface inflammation and damage, and neurosensory abnormalities [6,23]. Treatment options used in DED are moisturizing eye drops, antibiotics, and more aggressive immune modulators that can have serious side effects. However, specific treatment strategies to promote a healthy ocular surface microbiome, which in turn may alleviate DED, are currently unavailable, mainly due to the lack of knowledge of the composition of the ocular surface microbiome (OSM) and the role of its bacteria. In our study, we provided evidence that patients with DED have an altered composition of the OSM, and we also provided data on the tear proteome profile of patients with DED compared to non-DED individuals. 

### 3.1. The Role of the Human Ocular Surface Microbiome in Dry Eye Disease

We showed that the family *Propionibacteriaceae* and the species *Corynebacterium tuberculostearicum* were more abundant in DED patients compared to controls. Bacteria of the genus *Corynebacterium*, which are Gram-positive, aerobically growing rods, frequently colonize humans’ skin and mucosal surfaces. *Corynebacterium tuberculostearicum* is a lipophilic, catalase-positive, and CAMP-negative corynebacterial species only characterized in 2004 [24]. It has been shown that *Corynebacterium tuberculostearicum* elicits inflammation via the canonical NF-κB pathway and can upregulate the expression of TLR2, another inflammatory mediator [25]. *Propionibacteriaceae* are Gram-positive, anaerobic rods associated with human infections, including corneal infections, and are often found in acne vulgaris patients. Intradermal infections of *Propionibacteriaceae* generate ROS and nitric oxide and induce the synthesis of pro-inflammatory cytokines. Since an imbalance of these ROS and antioxidative tear proteins favors the development of tear film instability [18,19], as observed in DED, *Propionibacteriaceae* may be a key player in the interaction between the tear proteome and the OSM in DED. Moreover, it seems that both *Corynebacterium tuberculostearicum* and bacteria of the family *Propionibacteriaceae* are involved in inflammatory processes through the upregulation of pro-inflammatory cytokines that may be associated with DED.

In this study, we used whole-metagenome shotgun sequencing to characterize the OSM. Compared to 16s rRNA sequencing, the former allows the identification of viruses, archaea, and eukaryotes in addition to bacteria and a more reliable functional profiling [26]. Compared to controls, the conjunctiva of DED patients was decreased in genes of L-lysine biosynthesis, tetrapyrrole biosynthesis, 5-aminoimidazole ribonucleotide biosynthesis, and the super pathway of L-threonine biosynthesis. It has been shown that L-lysine controls viruses by interfering in the formation of viral capsid proteins and DNA [27]. Since more viruses are present in non-DED individuals, upregulated L-lysine biosynthesis may reflect a viral control in the OSM of these individuals.

Moreover, a competitive antagonism exists between L-lysine and L-arginine, an essential amino acid for some viruses [27]. Although further investigation is needed, the increased L-arginine biosynthesis in the conjunctiva of patients with tear hyperosmolarity may further support the above hypothesis that in patients with higher osmolarity levels (i.e., DED patients), more L-arginine is present as no suppressive L-lysine is needed for viral control. Although more investigation is needed, we speculate that L-lysine is important in maintaining the homeostasis of the OSM via the prevention of a viral overload, and its antagonist, L-arginine, is a marker for enhanced tear osmolarity in DED.

### 3.2. The Role of the Human Tear Proteome in Dry Eye Disease

Apart from the ocular surface microbiome, the tear proteome may be a key player in the pathogenesis of DED. Analyzing the tear proteome, we identified 112 proteins that differed between DED patients and controls. This is in keeping with the presumption that DED has a large inflammatory component, and many of these proteins were associated with innate immunity and the complement system. 

Phospholipase Ds belong to an important class of enzymes involved in inflammation. Their subtypes are present in the human ocular surface epithelium, where they may be involved in pterygium pathogenesis [28]. Since there was a lack of expression of the specific subtype phosphatidylinositol–glycan specific phospholipase D in the controls of our cohort, this enzyme may be a promising candidate for DED biomarker search with potential involvement in inflammatory processes, as observed in DED.

Mucin-16 is a membrane-associated mucin found on the apical membranes of the human cornea and conjunctiva epithelial cells. In addition to the classical mucin-associated functions, including maintaining hydration and lubrication of the epithelial surface and forming a disadhesive barrier, mucins have signaling functions. In human corneal epithelial cells with knocked down mucin-16 expression, the adherence of *Staphylococcus aureus* to epithelial cells, and the penetration of agents, such as the Rose Bengal dye used to diagnose DED, into epithelial cells were increased [29]. This indicates that mucin-16 may prevent the adhesion of both pathogens and agents. Moreover, several studies indicated that the extracellular domain of mucin-16 has been released into the tear film. It has been shown in vitro that this mechanism of shedding is induced via pro-inflammatory cytokines that are known to be upregulated in cases of dry eyes [30,31]. This is in agreement with the upregulation of mucin-16 in tears of DED patients compared to controls in our cohort and suggests a role of mucin-16 shedding in DED. The negative correlation between mucin-16 in tears and *Acinetobacter johnsonii* further supports this hypothesis since, in addition to pro-inflammatory cytokines, bacterial components may influence mucin-16 release into the tear film [30]. 

This correlation between tear components and members of the OSM reflects the influence and association of the tear proteome and the OSM in inflammatory processes, as observed in DED. To further substantiate the interaction of these systems in DED, we screened for tear proteins produced via bacteria of the OSM with a potential impact on DED. The three most abundant bacterial tear proteins were the chaperone protein DnaK, the arsenical resistance protein ArsH, and the helicase bacterial protein. The chaperone protein DnaK is a protein expressed via bacteria during stress. When infecting the host, the host’s defense mechanisms trigger stress in the pathogens, by which these pathogenic bacteria respond with a rapid acceleration in the rate of expression of heat shock as well as DnaK proteins. For example, the DnaK chaperone machinery of *Salmonella enterica* has been shown to be involved in the bacterial invasion of epithelial cells and might also be involved in the invasion of other tissues. Furthermore, it has been shown that the chaperone protein DnaK inhibitors display anti-bacterial activity [32].

The arsenical resistance protein ArsH is involved in the bacterial response to oxidative stress. Recent research has shown that ArsH can act as an organoarsenic reductase and thereby play a role in arsenic metabolism and toxicity in bacteria [33]. Unfortunately, very little is known about bacteria expressing the ArsH protein, mostly found in soil, in relation to the human microbiome. 

The helicase bacterial protein, found to originate from *Cutibacterium acnes* in our study, is another interesting protein that merits further discussion. Helicase proteins are dedicated enzymes that play a role in unwinding DNA duplexes to provide a single-stranded template to DNA polymerases for strand synthesis [34]. As the process of DNA replication is one of the most basic functions, this could be a prime target for antibiotic development. The helicase–primase interaction in bacteria is understudied and not yet a target for antibiotic development, in contrast to better-known DNA replication processes. Known antibiotics, like the bactericidal fluoroquinolone class of antibiotics that interferes with the DNA gyrase and topoisomerase IV activities, target the DNA replication machinery, but no other marketed drugs target other components [35]. All of these proteins may represent targets for novel therapeutic approaches for DED.

Limitations of this study include the limited number of patients and the lack of uniform diagnostic criteria for DED [36]. This is further complicated by the fact that there is a dissociation between signs and symptoms [37] and a patient’s perception of these symptoms [38]. According to the patient’s specific characteristics and disease severity, the sensitivity and specificity of the available diagnostic tools vary significantly [36]. Therefore, the most accurate diagnosis can be reached by combining the results of different tests as assessed in this study. In total, 80 patients were examined, but only the 10 patients with the highest DED score (DED patients) and the 10 patients with the smallest DED score (control group) were selected for group assignment and further analysis.

### 3.3. Conclusions

We performed a taxonomical and functional profiling of the OSM and a functional characterization of the tear proteome, revealing the importance of both systems and their associations in DED and facilitating the development of novel strategies for associated diseases.

## 4. Materials and Methods

### 4.1. Study Design and Recruitment

This study was reviewed and approved by the Ethics Committee of the Canton of Bern (ClinicalTrials.gov: NCT04656197). The procedures followed the tenets of the Declaration of Helsinki and the International Ethical Guidelines for Biomedical Research involving Human Subjects (Council for International Organizations of Medical Sciences).

The prospective observational (single-center) cross-sectional study was performed between October and December 2019. A total of 20 patients aged 60 and above attending routine follow-ups in daily clinical practice at the Department of Ophthalmology of the University Hospital Bern (Inselspital), Switzerland, were recruited. After receiving oral and written information, all participants gave written informed consent before study enrolment. Exclusion criteria were a history of recent (last 3 months) ocular surgery and systemic or topical antibiotics within the last three months. Smokers and participants wearing contact lenses or using systemic immunomodulators and corticosteroids or medical eye drops (except moisturizing eye drops) were excluded. After verifying that no exclusion criteria were met and written informed consent was given, one representative eye was randomly chosen for inclusion.

### 4.2. Assessment of Dry Eye Disease

The cohort was divided into two study groups: the dry eye disease group and the healthy control group. In order to assign the subjects to the study groups, objective and subjective assessment of DED was performed using the Ocular Surface Disease Index© (OSDI©, Allergan, Inc., Dublin, Irland) questionnaire, the TearLab Osmolarity Test, slit lamp examination, and a Schirmer’s Test II.

The OSDI© is a valid and reliable questionnaire to evaluate the severity of dry eye disease [14]. It consists of 12 questions assessing dry eye symptoms, vision-related function, and environmental triggers in the past week of the patient’s life.

To measure tear film osmolarity, the TearLab Osmolarity Test was performed according to the manufacturer’s protocol at https://www.tearlab.com/resources.net. Tear hyperosmolarity is defined by a referent of 316 mOsmol/L and values > 316 mOsmol/L and is indicative of DED [15]. 

Slit lamp examination was performed without fluorescein, as fluorescein may alter the microbial representation on the ocular surface. The examination was carried out by the same clinician for all study subjects to ensure consistency. The status of the eyelid margins, the conjunctiva, and the cornea were evaluated. Any present swelling of the eyelid was documented. Furthermore, redness and debris of the eyelid were assessed with a scoring range from 0 to 3 according to the rating scale used by Hosseini et al. [16]. Meibomian glands were examined for hyperemia, thickened lipid secretion, keratinization, and present chalazion, whereas conjunctivae were examined for swelling, erythema, papillae, and follicles. Erythemas were graded from 0 to 3 (0 = none: no injection present, 1 = mild: superficial localized injection, 2 = moderate: superficial general diffuse injection, and 3 = severe: superficial marked diffuse injection). Furthermore, whether the injection was conjunctival, pericorneal, ciliary, or mixed was documented. For corneal changes, a grading scale from 0 to 3 was applied (0 = normal, 1 = sparse density, 2 = moderate density, and 3 = high density and overlapped lesions).

Schirmer’s Test II was conducted to measure the baseline tear secretion under local anesthesia with 1% tetracaine (Théa, Schaffhausen, Switzerland) using tear strips (Haag-Streit, Köniz, Switzerland). A reading of less or equal to 10 mm wetting was considered dry eye.

To obtain an overall DED score for group assignment, the following grading system was applied: for the OSDI© questionnaire: severe = 1, moderate = 0.5, and mild = 0.25; TearLab Osmolarity: values > 316 mOsmol/L = 1; slit lamp examination: points were equal to the grading system; Schirmer’s Test: values ≤ 10 mm = 1. Participants with a DED score ≤ 6 were assigned to the control group, and participants with a score ≥ 16 were assigned to the DED group.

For a selected subgroup, meibography was performed using the LacryDiag Ocular Surface Analyzer (Quantel Medical, Cournon-d’Auvergne, France) according to the protocol described in [39].

### 4.3. Sample Collection

Wet tear strips from Schirmer’s Test II were collected in a 0.5 mL Protein LoBind tube (Eppendorf, Schönenbuch, Switzerland) punctured at the bottom with a cannula. This tube was then put into a larger 2 mL Protein LoBind tube (Eppendorf, Schönenbuch, Switzerland) and centrifuged at maximum speed for 5 min. The extracted tear fluid was stored at −80 °C until further analysis via nano liquid chromatography–tandem mass spectrometry (nLC-MS/MS; see below).

After assessment of DED, two separate sterile cotton swabs (Applimed SA, Châtel-St-Denis, Switzerland) were used to obtain swabs from the tarsal conjunctiva and lower eyelid margin, respectively. The swabs were then placed separately into a 2 mL DNA LoBind Tube (Eppendorf, Schönenbuch, Switzerland) and stored at 4 °C until further analysis on the same day. For negative controls, sterile cotton swabs without (*n* = 2) tetracaine and with one drop of 1% tetracaine (*n* = 2), respectively, were processed as a lid and conjunctival swabs.

### 4.4. DNA Extraction and Metagenomics DNA Sequencing

The DNA was extracted using the E.Z.N.A.^®^ MicroElute Genomic DNA Kit (Omega Bio-Tek, Norcross, GA, USA) according to the manufacturer’s protocol with an integrated RNA digestion step using 100 mg/mL RNAse (Qiagen, Hombrechtikon, Switzerland). DNA was stored at −20 °C until further analysis.

The DNA samples were brought to the Next Generation Sequencing Platform of the University of Bern, Switzerland, for metagenomic shotgun sequencing. The Nextera DNA Flex Library Preparation kit (Illumina, San Diego, CA, USA) was used for library preparation for sequencing following standard pipelines of the Illumina NovaSeq 6000 sequencing system. To exclude low-quality and human reads, the 150 bp paired-end reads were quality filtered using Trimmomatic v.0.32 [40] and mapped to the human reference genome hg19 using Bowtie2 v.2.2.4 [41]. For taxonomical annotation, the Metagenomic Phylogenetic Analysis tool v.2.6.0 (MetaPhlAn2) and the marker database v.20 [42] were applied with default settings. The relative abundances of each taxonomic unit were calculated using Bowtie2 for alignment, followed by normalization of the total number of reads in each clade by the nucleotide length of its marker. For functional annotation, the HMP (Human Microbiome Project [43]) Unified Metabolic Analysis Network (HUMAnN2 v.0.11.0 [44]) was used with default settings. To provide a functional annotation of the taxonomical profiles from MetaPhlAn2, HUMAnN2 was run for each sample separately as described in Zysset-Burri et al. [9], ending up in a set of pathways including their abundances.

### 4.5. Nano Liquid Chromatography–Tandem Mass Spectrometry (nLC-MS/MS)

Tear fluid was processed as described in (11). Protein in-gel digestion was performed according to the description in (37). An aliquot of 5 µL from each digest was analyzed via nLC-MS/MS on an instrumental setup consisting of an EASY-nLC 1000 chromatograph coupled with a QExactive HF mass spectrometer (ThermoFisher Scientific, Reinach, Switzerland) as described (11).

Mass spectrometry data were processed with MaxQuant/Andromeda version 1.6.1.0 [45] using default settings for peak detection, a strict trypsin cleavage rule, allowing up to three missed cleavages, variable oxidation on methionine, acetylation of protein N-termini, deamination of asparagine and glutamine, and fixed carbamidomethylation of cysteines, respectively. A match between runs was used with a retention time window of 0.7 min between neighboring gel fractions but not between samples. The SwissProt human protein sequence database (version 2019_02) was used to interpret fragment spectra with an initial mass tolerance of 10 ppm on the precursor and 20 ppm for fragment ions, respectively. Protein identifications were accepted if at least two razor peptides per protein group were identified at a 1% false discovery rate (FDR) cutoff.

### 4.6. Statistical Analysis

Demographics were compared among groups applying either Fisher’s exact test (for sex) or Welch’s *t*-test (for age, OSDI© score, osmolarity, slit lamp score, and Schirmer’s Test ll, Table 1). R software (version 3.6.1) was used to perform other analyses. To provide a global analysis of microbial and pathway abundances between groups via PCA, the R package *ade4* [46] was used. The PCA was performed using scaled values on the relative abundances of microbes (identified via MetaPhlAn2) and microbial pathways (identified via HUMAnN2). A visualization of the individual samples grouped by patients and controls as well as by lid and conjunctiva is provided in Figure 4A,B. A *p*-value for group separation was provided by applying a permutation multivariate analysis of variance (PERMANOVA) using the R package *vegan* [47] (with 1000 permutations). In order to examine whether relative abundances of microbial taxa and pathways were associated with clinical metadata and/or quantities of the tear proteins, the MaAsLin R package [48] was applied. Significant associations were considered below a q-value threshold of 0.25 and N ≠ 0 in at least 50% of the samples after adjusting for FDR (Benjamini–Hochberg).

## Figures and Tables

**Table 1:** Characteristics of study participants.

Feature	DED (*n* = 10)	CTRL (*n* = 10)	*p* Value DED vs. CTRL
Males (*n*)	7	8	1.0 ^1^
Age (years)	69.6 ± 11.3	69.7 ± 4.2	1.0 ^2^
OSDI© score	13.8 ± 12.4	7.8 ± 7.7	0.2 ^2^
Osmolarity (mOsmol/L)	306.3 ± 30.4	298.8 ± 15.0	0.5 ^2^
Slit lamp score	17.6 ± 1.6	2.9 ± 1.9	<0.0001 ^2^
Schirmer’s test ll (mm)	14.4 ± 7.0	16.7 ± 6.0	0.4 ^2^

^1^ Fisher’s exact test; ^2^ Welch’s *t*-test; data are mean ± SD; CTRL, controls; DED, dry eye disease; OSDI©, Ocular Surface Disease Index©.

**Table 2:** Relationship between tear osmolarity and L-arginine biosynthesis in the conjunctiva.

Osmolarity (mOsmol/L)	L-arginine Biosynthesis (%)
267	0
286	0
288	0
288	0
289	1.58
289	0
295	0
297	0
300	0
305	0
312	0
335	1.76
354	1.62
365	1.56

Tear hyperosmolarity is defined via a referent of 316 mOsmol/L.

**Table 3:** Bacterial proteins in human tears.

Taxonomy	Protein	Nb. of Samples
*Acinetobacter johnsonii*	Arsenical resistance protein ArsH	18
*Acinetobacter* sp.	DNA-binding response regulator	2
*Agrobacterium radiobacter*	Polyribonucleotide nucleotidyltransferase	2
*Agrobacterium radiobacter*	Molybdopterin biosynthesis protein	1
*Agrobacterium* sp.	Chaperone protein DnaK	19
*Agrobacterium* sp.	Anthranilate synthase	8
*Agrobacterium* sp.	Uncharacterized protein	3
*Agrobacterium* sp.	Phage tail protein	1
*Agrobacterium* sp.	Membrane fusion protein (MFP)	7
*Agrobacterium* sp.	DEAD/DEAH box helicase	8
*Campylobacter ureolyticus*	Cytochrome c biogenesis protein	4
*Corynebacterium accolens*	Bifunctional RNase H/acid phosphatase	15
*Cutibacterium acnes*	Helicase	17
*Cutibacterium acnes*	Ribonuclease Y	10

Number of samples the protein was detected in (with a total number of *n* = 20).

## Data Availability

The datasets supporting the conclusions of this article are available in the European Nucleotide Archive under the accession number PRJEB38989.

## References

[1] Dana R., Bradley J.L., Guerin A., Pivneva I., Stillman I.O., Evans A.M., Schaumberg D.A. (2019). Estimated Prevalence and Incidence of Dry Eye Disease Based on Coding Analysis of a Large, All-age United States Health Care System. Am. J. Ophthalmol..

[2] Stapleton F., Alves M., Bunya V.Y., Jalbert I., Lekhanont K., Malet F., Na K.S., Schaumberg D., Uchino M., Vehof J. (2017). TFOS DEWS II Epidemiology Report. Ocul. Surf..

[3] Zhang X., Jeyalatha M. V., Qu Y., He X., Ou S., Bu J., Jia C., Wang J., Wu H., Liu Z. (2017). Dry Eye Management: Targeting the Ocular Surface Microenvironment. Int. J. Mol. Sci..

[4] Uchino M., Schaumberg D.A. (2013). Dry Eye Disease: Impact on Quality of Life and Vision. Curr. Ophthalmol. Rep..

[5] Craig J.P., Nichols K.K., Akpek E.K., Caffery B., Dua H.S., Joo C.K., Liu Z., Nelson J.D., Nichols J.J., Tsubota K. (2017). TFOS DEWS II Definition and Classification Report. Ocul. Surf..

[6] Galletti J.G., Guzman M., Giordano M.N. (2017). Mucosal immune tolerance at the ocular surface in health and disease. Immunology.

[7] Zinkernagel M.S., Zysset-Burri D.C., Keller I., Berger L.E., Leichtle A.B., Largiader C.R., Fiedler G.M., Wolf S. (2017). Association of the Intestinal Microbiome with the Development of Neovascular Age-Related Macular Degeneration. Sci. Rep..

[8] Zysset-Burri D.C., Keller I., Berger L.E., Largiader C.R., Wittwer M., Wolf S., Zinkernagel M.S. (2020). Associations of the intestinal microbiome with the complement system in neovascular age-related macular degeneration. NPJ Genom. Med..

[9] Zysset-Burri D.C., Keller I., Berger L.E., Neyer P.J., Steuer C., Wolf S., Zinkernagel M.S. (2019). Retinal artery occlusion is associated with compositional and functional shifts in the gut microbiome and altered trimethylamine-N-oxide levels. Sci. Rep..

[10] Moon J., Choi S.H., Yoon C.H., Kim M.K. (2020). Gut dysbiosis is prevailing in Sjogren’s syndrome and is related to dry eye severity. PLoS ONE.

[11] Napolitano P., Filippelli M., Davinelli S., Bartollino S., dell’Omo R., Costagliola C. (2021). Influence of gut microbiota on eye diseases: An overview. Ann. Med..

[12] Horai R., Caspi R.R. (2019). Microbiome and Autoimmune Uveitis. Front. Immunol..

[13] Li Z., Zhu H., Zhang L., Qin C. (2018). The intestinal microbiome and Alzheimer’s disease: A review. Anim. Model. Exp. Med..

[14] Opazo M.C., Ortega-Rocha E.M., Coronado-Arrazola I., Bonifaz L.C., Boudin H., Neunlist M., Bueno S.M., Kalergis A.M., Riedel C.A. (2018). Intestinal Microbiota Influences Non-intestinal Related Autoimmune Diseases. Front. Microbiol..

[15] Pascal V., Pozuelo M., Borruel N., Casellas F., Campos D., Santiago A., Martinez X., Varela E., Sarrabayrouse G., Machiels K. (2017). A microbial signature for Crohn’s disease. Gut.

[16] Silverman G.J. (2019). The microbiome in SLE pathogenesis. Nat. Rev. Rheumatol..

[17] Zarate-Blades C.R., Horai R., Mattapallil M.J., Ajami N.J., Wong M., Petrosino J.F., Itoh K., Chan C.C., Caspi R.R. (2017). Gut microbiota as a source of a surrogate antigen that triggers autoimmunity in an immune privileged site. Gut Microbes.

[18] Zysset-Burri D.C., Schlegel I., Lincke J.B., Jaggi D., Keller I., Heller M., Lagache S.B., Wolf S., Zinkernagel M.S. (2021). Understanding the Interactions between the Ocular Surface Microbiome and the Tear Proteome. Investig. Ophthalmol. Vis. Sci..

[19] Seen S., Tong L. (2018). Dry eye disease and oxidative stress. Acta Ophthalmol..

[20] Heidari M., Noorizadeh F., Wu K., Inomata T., Mashaghi A. (2019). Dry Eye Disease: Emerging Approaches to Disease Analysis and Therapy. J. Clin. Med..

[21] Huang da W., Sherman B.T., Lempicki R.A. (2009). Bioinformatics enrichment tools: Paths toward the comprehensive functional analysis of large gene lists. Nucleic Acids Res..

[22] Huang Y., Yang B., Li W. (2016). Defining the normal core microbiome of conjunctival microbial communities. Clin. Microbiol. Infect..

[23] Stern M.E., Schaumburg C.S., Pflugfelder S.C. (2013). Dry eye as a mucosal autoimmune disease. Int. Rev. Immunol..

[24] Feurer C., Clermont D., Bimet F., Candrea A., Jackson M., Glaser P., Bizet C., Dauga C. (2004). Taxonomic characterization of nine strains isolated from clinical and environmental specimens, and proposal of *Corynebacterium tuberculostearicum* sp. nov. Int. J. Syst. Evol. Microbiol..

[25] Altonsy M.O., Kurwa H.A., Lauzon G.J., Amrein M., Gerber A.N., Almishri W., Mydlarski P.R. (2020). *Corynebacterium tuberculostearicum*, a human skin colonizer, induces the canonical nuclear factor-kappaB inflammatory signaling pathway in human skin cells. Immun. Inflamm. Dis..

[26] Jovel J., Patterson J., Wang W., Hotte N., O’Keefe S., Mitchel T., Perry T., Kao D., Mason A.L., Madsen K.L. (2016). Characterization of the Gut Microbiome Using 16S or Shotgun Metagenomics. Front. Microbiol..

[27] Pedrazini M.C., da Silva M.H., Groppo F.C. (2022). L-lysine: Its antagonism with L-arginine in controlling viral infection. Narrative literature review. Br. J. Clin. Pharmacol..

[28] Tong L., Li J., Chew J., Tan D., Beuerman R. (2008). Phospholipase D in the Human Ocular Surface and in Pterygium. Cornea.

[29] Blalock T.D., Spurr-Michaud S.J., Tisdale A.S., Heimer S.R., Gilmore M.S., Ramesh V., Gipson I.K. (2007). Functions of MUC16 in corneal epithelial cells. Investig. Ophthalmol. Vis. Sci..

[30] Paulsen F., Jager K., Worlitzsch D., Brauer L., Schulze U., Schafer G., Sel S. (2008). Regulation of MUC16 by inflammatory mediators in ocular surface epithelial cell lines. Ann. Anat..

[31] Blalock T.D., Spurr-Michaud S.J., Tisdale A.S., Gipson I.K. (2008). Release of membrane-associated mucins from ocular surface epithelia. Investig. Ophthalmol. Vis. Sci..

[32] Liebscher M., Jahreis G., Lucke C., Grabley S., Raina S., Schiene-Fischer C. (2007). Fatty acyl benzamido antibacterials based on inhibition of DnaK-catalyzed protein folding. J. Biol. Chem..

[33] Sedlacek V., Kryl M., Kucera I. (2022). The ArsH Protein Product of the Paracoccus denitrificans ars Operon Has an Activity of Organoarsenic Reductase and Is Regulated by a Redox-Responsive Repressor. Antioxid.

[34] Strycharska M.S., Arias-Palomo E., Lyubimov A.Y., Erzberger J.P., O’Shea V.L., Bustamante C.J., Berger J.M. (2013). Nucleotide and partner-protein control of bacterial replicative helicase structure and function. Mol. Cell.

[35] Soultanas P. (2005). The bacterial helicase-primase interaction: A common structural/functional module. Structure.

[36] Elhusseiny A.M., Khalil A.A., El Sheikh R.H., Bakr M.A., Eissa M.G., El Sayed Y.M. (2019). New approaches for diagnosis of dry eye disease. Int. J. Ophthalmol..

[37] Sullivan B.D., Crews L.A., Messmer E.M., Foulks G.N., Nichols K.K., Baenninger P., Geerling G., Figueiredo F., Lemp M.A. (2014). Correlations between commonly used objective signs and symptoms for the diagnosis of dry eye disease: Clinical implications. Acta Ophthalmol..

[38] van Setten G., Labetoulle M., Baudouin C., Rolando M. (2016). Evidence of seasonality and effects of psychrometry in dry eye disease. Acta Ophthalmol..

[39] Ward C.D., Murchison C.E., Petroll W.M., Robertson D.M. (2021). Evaluation of the Repeatability of the LacryDiag Ocular Surface Analyzer for Assessment of the Meibomian Glands and Tear Film. Transl. Vis. Sci. Technol..

[40] Bolger A.M., Lohse M., Usadel B. (2014). Trimmomatic: A flexible trimmer for Illumina sequence data. Bioinformatics.

[41] Langmead B., Salzberg S.L. (2012). Fast gapped-read alignment with Bowtie 2. Nat. Methods.

[42] Segata N., Waldron L., Ballarini A., Narasimhan V., Jousson O., Huttenhower C. (2012). Metagenomic microbial community profiling using unique clade-specific marker genes. Nat. Methods.

[43] The Human Microbiome Project Consortium (2012). A framework for human microbiome research. Nature.

[44] Abubucker S., Segata N., Goll J., Schubert A.M., Izard J., Cantarel B.L., Rodriguez-Mueller B., Zucker J., Thiagarajan M., Henrissat B. (2012). Metabolic reconstruction for metagenomic data and its application to the human microbiome. PLoS Comput. Biol..

[45] Cox J., Mann M. (2008). Maxquant Enables High Peptide Identification Rates, Individualized P.P.B.-Range Mass Accuracies and Proteome-Wide Protein Quantification. Nat. Biotechnol..

[46] Dray S., Dufour A.B. (2007). The ade4 Package: Implementing the Duality Diagram for Ecologists. J. Stat. Softw..

[47] Anderson M.J. (2001). A new method for non-parametric multivariate analysis of variance. Austral Ecol..

[48] Morgan X.C., Tickle T.L., Sokol H., Gevers D., Devaney K.L., Ward D.V., Reyes J.A., Shah S.A., LeLeiko N., Snapper S.B. (2012). Dysfunction of the intestinal microbiome in inflammatory bowel disease and treatment. Genome Biol..

